# Laser ablation plasma expansion using microwaves

**DOI:** 10.1038/s41598-023-41208-z

**Published:** 2023-08-25

**Authors:** Yuji Ikeda, Joey Kim Soriano, Hironori Ohba, Ikuo Wakaida

**Affiliations:** 1 i-Lab., Inc., #213 KIBC Bldg., 5-5-2 Minatojima-Minami, Chuo, Kobe, 650-0047 Japan; 2 Collaborative Laboratory for Advanced Decommissioning Science, Japan Atomic Energy Agency (JAEA), 2-4 Shirakata, Tokai-Mura, Naka-Gun, Ibaraki, 319-1195 Japan; 3 National Institutes for Quantum Science and Technology (QST), 2-4 Tokai-Mura, Naka-Gun, Ibaraki, 319-1106 Japan; 4 Collaborative Laboratory for Advanced Decommissioning Science, Japan Atomic Energy Agency (JAEA), 790-1 Motooka, Tomioka-Machi, Futaba-Gun, Fukushima, 979-1151 Japan

**Keywords:** Laser-produced plasmas, Spectrophotometry

## Abstract

This study explores the potential of utilizing microwaves to sustain the expansion of transient laser ablation plasma of Zr target. By application of microwaves on the plasma, we observe a significant enhancement with a two to three order of magnitude increase in the plasma emission intensity, and 18 times increase in the plasma’s spatial volume. We investigate the temperature change of the plasma and observe that it decreases from 10,000 K to approximately 3000 K. Electron temperature decreased with volume expansion owing to increased surrounding air interaction, while the plasma can be sustained in air using microwaves. The increase in electron temperature during temperature drop is indicative of non-equilibrium plasma. Our results emphasize the contribution of microwaves in promoting enhanced emission and plasma formation at controlled, low temperature, thereby demonstrating the potential of microwaves to enhance the accuracy and performance of laser-induced breakdown spectroscopy. Importantly, our study suggests that microwaves could also mitigate the generation of toxic fumes and dust during ablation, a critical benefit when handling hazardous materials. The system we've developed is highly valuable for a range of applications, notably including the potential to reduce the possible emergence of toxic fumes during the decommissioning of nuclear debris.

## Introduction

Laser ablation plasma generates a breakdown plasma that rapidly expands in space and dissipates within nanoseconds to microseconds, finding widespread applications in instrumentation, medicine, and industry^1, 2^. It involves exposing a sample target to a pulsed laser, resulting in plasma with characteristics that can vary significantly owing to various factors, including self-absorption, reflection, and cooling. The control over the plasma characteristics can be demonstrated by the plasma emissions.

Analytical applications using laser-induced breakdown spectroscopy (LIBS)^3–13^ have proven to be a powerful tool in science and industry. The amount of plasma emission can vary greatly depending on the environmental conditions under which the plasma is generated, and its properties can be controlled for a range of applications such as low-pressure semiconductor manufacturing equipment^14, 15^, space applications in a vacuum, elemental analysis^16–18^, high-pressure internal combustion engines^19^, and deep-sea applications^20^. However, ablation plasmas are typically limited in their expansion due to system constraints, such as volume size change and plasma lifetime^21^, limitations that are being addressed by microwave-enhanced LIBS by combining microwaves and pulsed lasers resulting in a significantly improved performance of the system^21–32^. The emission intensity of the plasma is significantly enhanced by microwave superposition because microwave energy can sustain the plasma for a much longer period, allowing for more emission events to occur^15, 22, 31, 33–46^. In addition, the spatial volume of the plasma is expanded by two orders of magnitude, which further increases the amount of light emitted and detected by the system. This is critical for applications that require high sensitivity because even negligible changes in the amount of light emitted can significantly affect the accuracy of the measurement.

The laser-induced plasma in LIBS can exist in either equilibrium or nonequilibrium states^47–49^. It is crucial to understand the differences between equilibrium and nonequilibrium plasmas in LIBS to develop a more reliable and accurate analytical method as non-equilibrium plasma can result in enhanced or suppressed emission intensities of certain atomic or molecular lines^50, 51^. In the context of microwave-enhanced LIBS, we have observed distinct non-equilibrium characteristics, particularly in the rotational and vibrational temperatures. The rotational and vibrational temperatures^21^were measured to elucidate the rapid change in ablation plasma characteristics, indicating that plasma volume expansion leads to a decrease in vibrational temperature from 12,000 K to approximately 2200 K within 1 ms^21^. While many other processes are responsible or plasma expansion and it will not always be accompanied by a temperature drop, we theorized that the plasma expansion and temperature drop during the microwave-expansion and sustainment period (few microseconds after ablation) is caused by the increased interaction between the plasma and surrounding air atmosphere. The contribution of shock waves in the laser ablation process in microwave-couple ablation is considered insignificant due to the time delay between the laser firing and the penetration of microwaves into the laser-induced plasma. This delay occurs because the microwaves have to wait for the laser-induced plasma density to decrease below the critical density (~ 10^10^ to 10^11^ cm^-3^) required for microwave penetration. For a microwave radiation frequency of 2.45 GHz, this critical density is typically in the order of 7 × 10^10^ cm^−3^^52^. During the sustainment of plasma in air, the electrons can be accelerated and maintained at a certain level during the microwave injection period. The physics of microwave-enhanced plasma can be demonstrated by comparing plasma temperatures and its sustainment in air.

Previous studies have demonstrated the effectiveness of microwave-enhanced LIBS in increasing the plasma emission intensity by two to three orders of magnitude, without significant crater enlargement^23, 53–55^. Various samples, including Al, Al_2_O_3_, Gd, Ce, Pb, Cr, and Zr have been optimized for microwave oscillation conditions, and Zr and Zr oxides have also been determined to increase the plasma emission intensity^27, 56^. Although the plasma generated by the laser and expanded plasma maintained by the microwave may have different characteristics, both exhibit nonequilibrium temperatures, as evidenced by non-equal rotational and vibrational temperatures^24, 30^.

Our target application in using microwave-enhanced LIBS is on nuclear fuel debris analysis for the decommissioning effort at the Fukushima Daiichi Nuclear Power Station in Japan^31^. As the inevitable power losses in remote LIBS and high radiation-induced light absorption persists, the microwaves ensure sustained and large ablations even at threshold ablation energies. To prepare for the actual nuclear debris analysis, we examine various debris-related materials, including concrete and stainless steel from the building structure, Zirconium and Zirconia from the fuel rod tunnels, Gd from the fuel rod cladding, and Ce as a surrogate for Uranium. In this study, we used Zr as a sample for laser ablation plasma.

In the standard generation process of the laser-only induced process, the plasma volume expansion and emission increase are primarily driven by the rapid heating and vaporization of the target material upon laser irradiation. In microwave-assisted laser ablation plasma, the volume expansion is accompanied by a temperature drop as the microwaves influence plasma dynamics. In this study, we employed microlasers, which are compact laser systems with small, rectangular YAG crystal (dimensions 3.0 × 3.0 × 10.0 mm^3^) to generate the laser-induced breakdown plasma of Zirconium and Zirconia targets for microwave-enhanced LIBS analysis^23^. We wanted to emphasize that the plasma temperature sustained in air differed significantly from the laser-induced ablation, as the plasma expansion is accompanied by a temperature drop suggesting a non-equilibrium state. In this state, the electron temperature measured through OI emissions decreased, eventually leading to a non-equilibrium plasma state which remained constant as it was sustained by the microwaves. These results address fundamental questions regarding the effects of microwaves on ablation, plasma temperature, and ionization dynamics.

## Experimental system and plasma visualization

**Figure 1:**
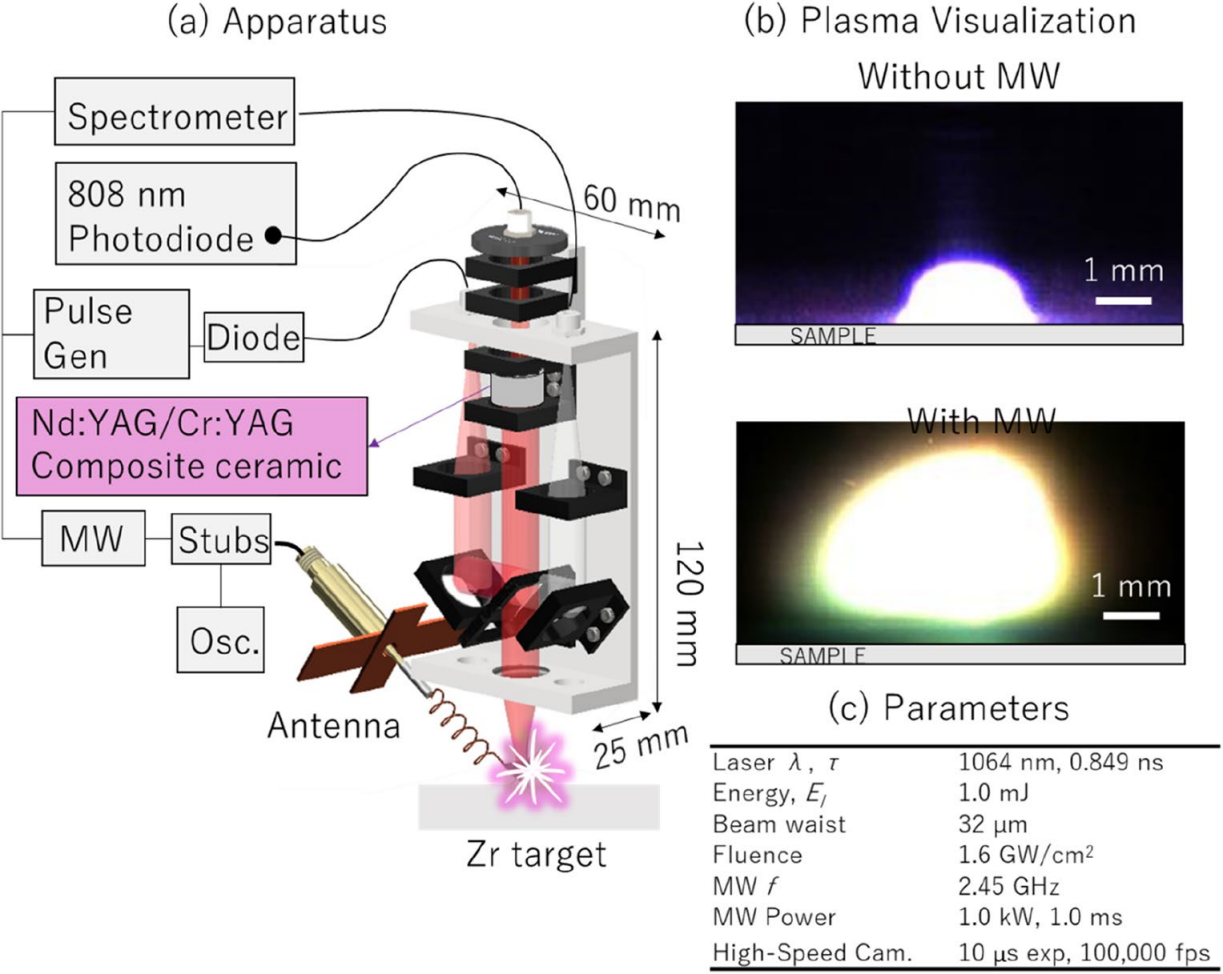
Experimental setup for microwave-enhanced laser-induced breakdown spectroscopy (LIBS) with micro-laser and the effects of microwaves on plasma formation. The elements depicted in the figure include three-tuber stubs for impedance matching (Stub), a pulse generator (Pulse Gen), a microwave generator (MW), an oscilloscope (Osc), and a photodiode (Diode).

Figure 1a illustrates the experimental setup of microwave-enhanced laser-induced breakdown spectroscopy (LIBS) with a microlaser^38^. The term “microlaser”, or microchip laser, describes the compact size and portability of the laser system. It is specifically designed to generate intense laser pulses for ablation and plasma formation on the target material. The microlaser utilized in the experiment is a composite ceramic (JOLD-120-QPXF-2P iTEC; JENOPTIK, FRG) that is end-pumped by an 808 nm laser diode powered by a 120 A, 200 W power supply (PS; PLWB168; UNITAC, Japan). The quasi-continuous wave is transmitted into the Nd:YAG of the microlaser and the Cr + 4:YAG acts as a saturable absorber, enabling passive Q-switching (> 60 μs) and emitting instantaneous laser pulses with a 1.0 mJ laser energy (849 ps pulse width and 1064 nm wavelength)^57, 58^. The composite ceramic and optical elements are housed in a 60 mm × 120 mm × 900 mm aluminum case. The laser output is transmitted into the beam splitter and InGaAs detector (DET08C/M; 800–1700 nm, bandwidth 5 GHz; Thorlabs, USA) with electrical pulses into the pulse generator, and this triggers microwaves and spectrometers. The same InGaAs detector was utilized directly to measure the laser pulse width, which was determined to be 0.849 ns. The 2.45 GHz microwaves was introduced by the helical coil with cross-reflector plates^26^. To minimize the reflected power, we employed an impedance tuner (three-stub tuner, Maury Microwave, USA) and monitored the power using power sensors of the directional coupler (440,000 series, Connecticut Microwave Corp, USA).

The plasma emissions were analyzed using a double echelle-type spectrometer (λ/150,000, Super Damon, LTB, Germany) and a single type-echelle spectrometer (λ/50,000, EMU 120/65, Catalina Scientific, Arizona). The exposure time and gate width used in both spectrometers was 1.0 ms. The Super Demon double echelle-type spectrometer, due to its higher resolution, was used for time-series signal-to-noise ratio (SNR) measurements. However, this spectrometer has a narrow spectral range, thus, we used the Catalina echelle spectrometer for temperature measurements. Despite its lower resolution, this spectrometer has a broader spectral range, allowing us to capture the several other emissions aside from the Zr emissions.

Figure 1b shows the plasma formation on Zr metal samples with and without microwave irradiation. An ultrafast camera (Fastcam SA-Z, Photron, UK) was used to visualize plasma formation with the same synchronized signal as the laser firing. At 100,000 fps with an exposure time of 10 μs, a 720 × 380 pixels^2^ measurement area was captured. In all experimental conditions, a Tamron 180 mm AF macro lens (Saitama, Japan) was fixed at an aperture value of F/3.5 (representing the f-number, which is the ratio of the lens's focal length to the diameter of the entrance pupil) and a zoom ratio of 1:10. The images displayed were the largest observed plasma at 0 μs without microwaves and 1000 μs with microwaves.

### Plasma visualization

To determine the plume circumference, we utilized image J analysis software and standard edge detection and pixel counting protocols. We then approximated the volume of the plasma as a hemispherical plume using the plume circumference. The estimation of the plasma plume volume as a hemisphere based on the plume circumference, while simplification, is a common approach in plasma physics, and we believe it's a valid first-order approximation in our case. This is, of course, dependent on the assumption that plasma expands symmetrically, which seems reasonable given our experimental setup and observations. It results in ablation volume significantly increases from 6.8 ± 0.1 to 125 ± 8 mm^3^ with the application of microwaves. The magnification is approximately 18 times, which is higher than the previously reported magnification of approximately ten times^24^. The observed plasma expansion of up to 18 times, compared to the previously reported 10 times, could be explained by two primary factors: microwave and laser parameters. When microwave parameters are held constant, the initial plasma size significantly impacts the enhancements, where larger initial sizes are likely to absorb more microwave energy, leading to more substantial expansions^29^. Nonetheless, we hypothesize that there is an optimal plasma size threshold, beyond which the efficiency of microwave-induced expansion could diminish. This hypothesis, however, requires further investigation for validation.

**Figure 2:**
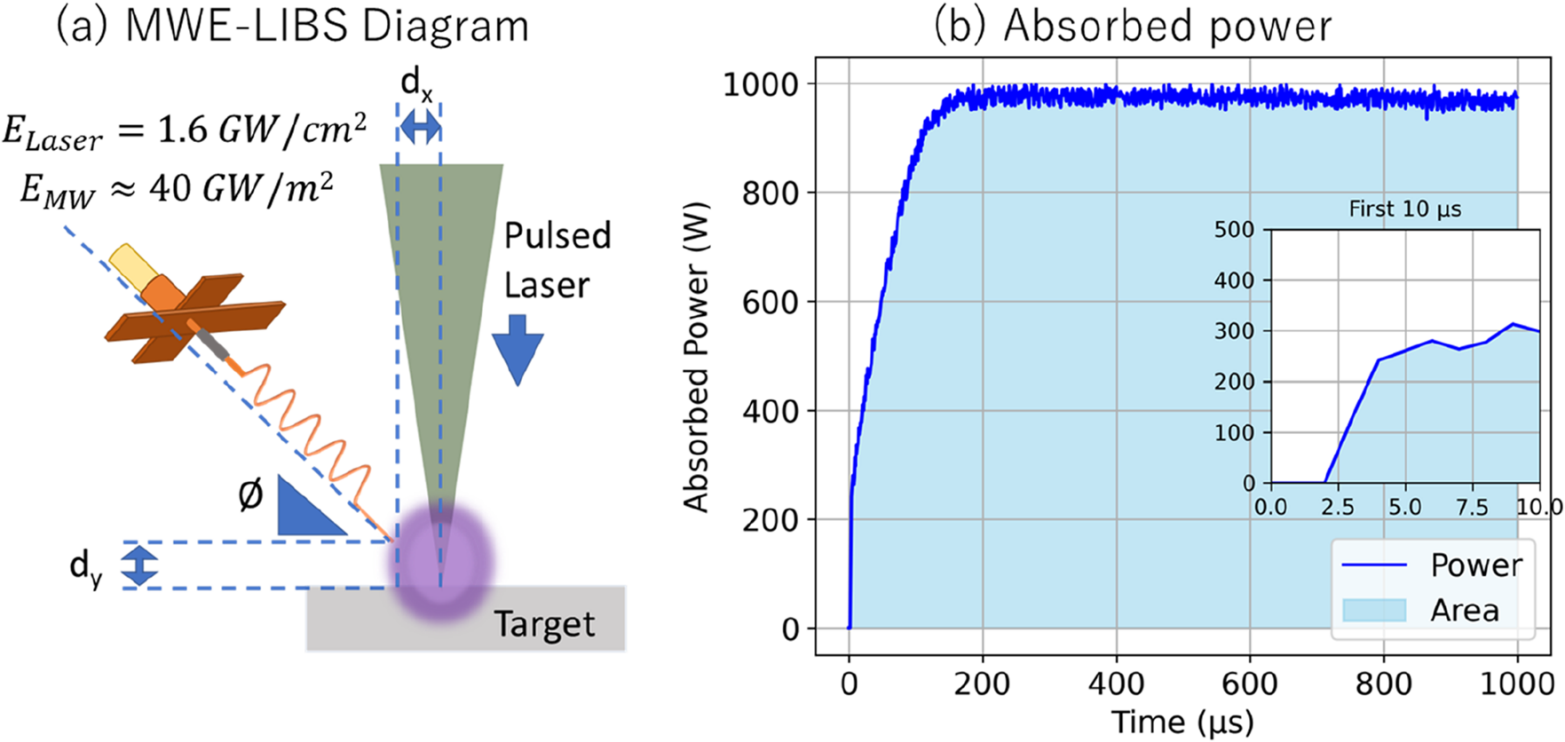
(**a**) The detailed diagram of the microwave antenna at *d*_*x*_ = *d*_*y*_ = 0.1 mm and Ø = 30◦. (**b**) The absorbed power plot was used to approximate the actual absorbed microwave power density by the enlarged plasma.

The superimposition of microwaves and laser-induced ablation functions as a near-field coupling system where the reactive near-field region is centered around the target area in close proximity to the antenna. In Fig. 2a, the microwave antenna position details are shown with values *d*_*x*_ = *d*_*y*_ = 0.1 mm and Ø = 30◦. A microwave power density of approximately 40 GW/m^2^, which corresponds to the maximum electric field of the antenna derived from HFSS simulation in reference and the radiation by the Poynting vector (E^2^/n, where E represents the electric field and n represents the intrinsic impedance), is delivered at a range of 0.1 mm from the target. This high-power density results to a significant enhancement of the laser-induced energy, initially at 1.6 GW/cm^2^. In the absorbed power measurements in Fig. 2b, the reflected power is subtracted from the forward power in realtime using the oscilloscope (DSO-X-3024A, AgilentTechnologies, Colorado, USA). We observed that the laser ablation plasma absorbs a significant amount of power at 0.981 J. During the initial 100 μs, the absorbed power is minimal, due to the high density of the laser ablation plasma, which impedes microwave propagation. During this period, we can only approximate and hypothesize that the plasma density is greater than critical plasma density of 7 × 10^10^ cm^−3^. Nevertheless, this absorbed power gradually escalates, reaching a maximum of 97.8% at around 60 μs. Factoring in both the absorbed power and our estimation of the plasma volume, we've calculated that the power density absorption by the plasma only approximates to 7.9 kW/cm^3^. It is hypothesized that this concentration of microwave energy may also influence the volume of fumes produced during the ablation process.

## Results and discussion

### Microwave superimposition in the ablation process

**Figure 3:**
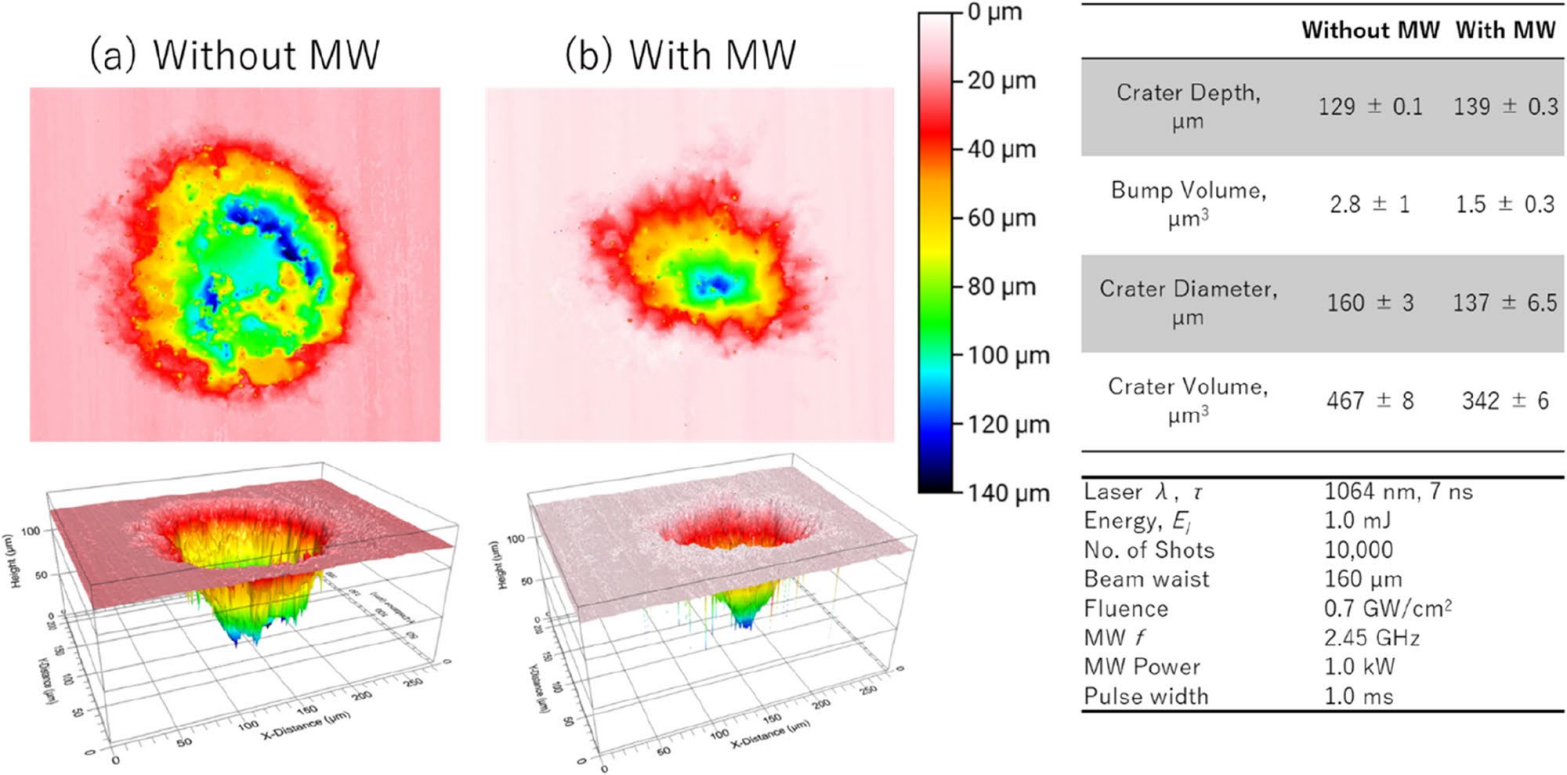
Laser-induced ablation of Zr oxide-based ceramic samples: changes in crater diameter and side bump characteristics both (**a**) without and (**b**) with microwave application. The relative standard errors were based on uncertainty of the reference surface which are measured by (i) standard deviation of the surface roughness for the depth distance, bump volume, and crater volume and (ii) standard deviation of the minimum and maximum diameter.

Figure 3 illustrates the outcome of laser-induced ablation conducted on the Zr oxide-based ceramic samples. The ablation procedure employed a laser energy of 1.0 mJ and microwave power of 1.0 kW. A three-dimensional (3D) microscopic camera (VHX-970FN, Keyence, Japan) with a resolution of 0.149 µm was utilized to capture the process and examine the surface ablation characteristics. The high melting temperatures of the Zr oxide-based ceramic samples, which reached 3,813 K, resulted in the formation of basin-like cavity profiles with side bumps that were easily discernible as shown in Fig. 3a. The point of origin in the crater’s depth is represented by a plain white color at the zero-micrometer mark, while the surrounding flat surface exhibits a range of light to dark pink colors, indicating the presence of elevated side bumps surrounding the crater. When microwaves were applied as shown in Fig. 3b, the diameter of the crater decreased, as the region near the antenna underwent less ablation. The volume of the side bump decreased; however, the depth increased. We speculated that microwaves contribute to the reduction of ablation crater volumes, which could in turn minimize the production of fumes and dust. This assumption seems to present an apparent contradiction with regards to the relationship between crater formation and plasma expansion where a more extensive material breakdown would be required to facilitate plasma expansion. To address this apparent discrepancy, we propose a hypothesis that the microwave-assisted breakdown of the side bumps facilitates plasma expansion. This hypothesis aligns with observed phenomena, as the plasma expansion is primarily outward and doesn’t infiltrate the crater's interior. Given our experimental setup, the laser's focal point is roughly located at the surface level and the focal depth is ~ 5 mm. Hence, with each succeeding laser shot, the ablation of materials slowly increase as laser drilling continues with increased laser shot.

In the absence of microwaves, a phenomenon known as plasma confinement occurs with an increase in the number of laser shots, leading to an enlargement of the crater’s volume. This observation is supported by the findings of Hernandez II et al., (2022)^59^, who reported an exponential increase in cavity size and depth with increased laser shots but eventually slows down due to plasma shielding effect. In the presence of microwaves, the enhancement is channeled upwards, inhibiting the progression of cavity enlargement. This unique mechanism allows microwaves to influence the plasma's depth and expansion. However, keep in mind that the microlaser device used is limited to the laser energy of 1 mJ. For larger laser energy, almost insignificant effects of microwave to ablation crater was observed on alumina surface^21^.

The recent results shows the potential of microwaves to reduce the ablation crater volume. The reason behind needs further investigation including microwave-induce breakdown and melting in the side bumps of the ablation with increased number of laser shots. This information is particularly significant in the context of dealing with toxic samples such as those encountered in our target application of nuclear debris decommissioning.

### Spectrum analysis

**Figure 4:**
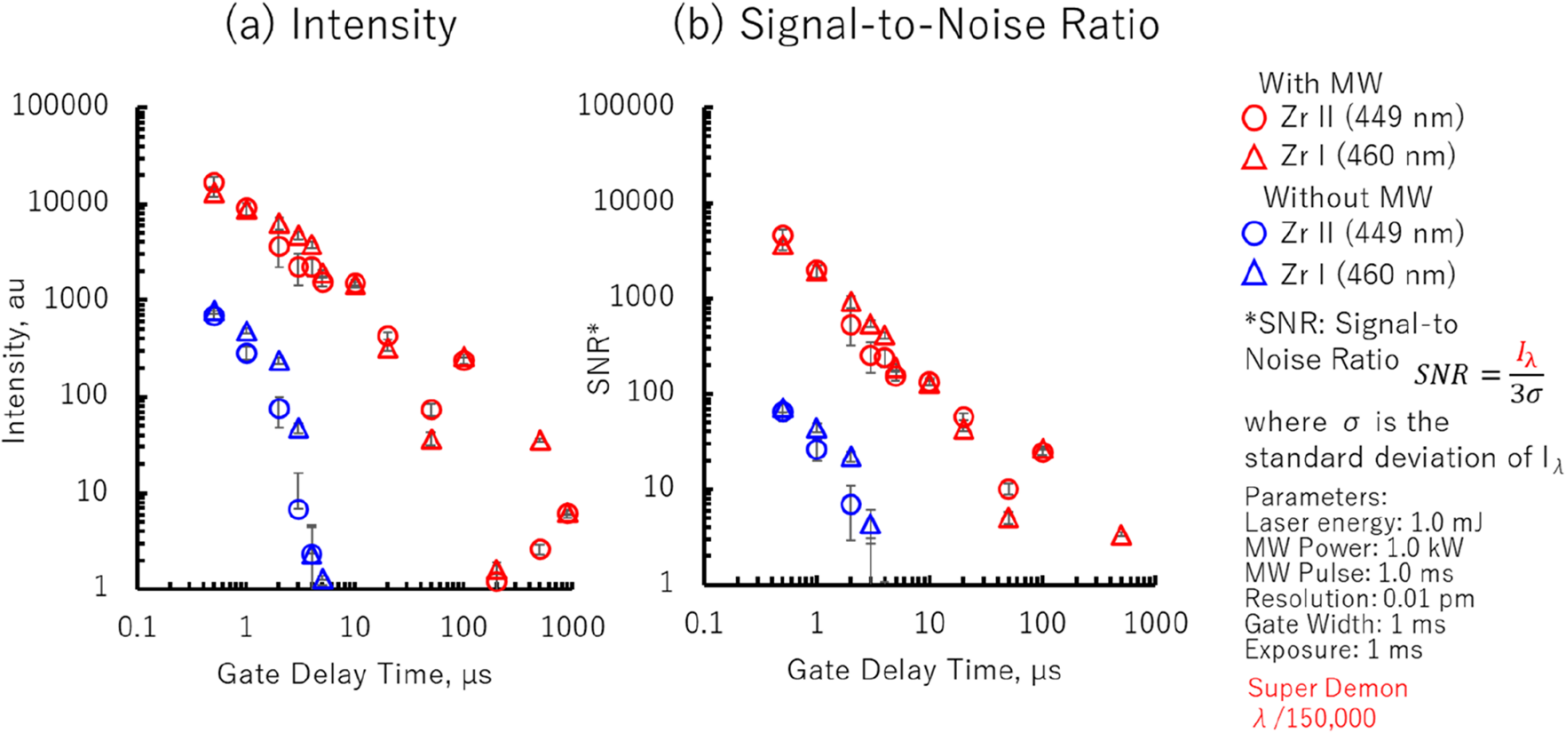
Effects of microwaves on the ablation plasma’s (**a**) emission intensity and (**b**) signal-to-noise ratio (SNR) of Zr samples.

Figure 4 illustrates the plasma emission intensities of atomic Zr I (460 nm) and ionic Zr II (449 nm) emissions at varying gate delays. The red symbols representing the results of the standard LIBS system with superimposed microwaves and blue symbols representing the results without microwaves. As the we are interested with nuclear debris applications, the Zr I and Zr II lines may interfere with Uranium emissions especially within the 420–470 nm range. Figure 4a shows the plasma emission intensity, whereas the Fig. 4b illustrates the signal-to-noise ratio (SNR) calculated as a ratio to the background light. Both figures illustrate an enhancement effect of approximately two–three orders of magnitude in the presence of microwaves.

Without microwaves, the ablation plasma disappears approximately 10 μs after the laser irradiation. However, when microwaves are superimposed, the ablation plasma is sustained in space for a longer duration. The plasma is maintained during the microwave input period, as indicated by the time change of the emission intensity and SNR value of the characteristic spectrum. The trends are consistent for the neutral and ion emissions, and the enhancement effect of the microwaves is approximately two to three-orders-of-magnitude.

Several other reports suggest an improved SNR only by a factor in the single or double digits as opposed to the two to three-orders-of-magnitude as other factors affect SNR including increased background noise by microwaves. Recently, we found that the peak SNR for conditions without microwaves occurs within the first microsecond after laser firing. This is a detail we've previously overlooked, as our focus has been primarily on plasma emissions coupled with microwaves. To be clear, the enhancements of two to three orders of magnitude only pertain to the same emissions at a gate delay between 1 and 10 μs. However, when comparing the optimal SNR under conditions with or without microwaves, the increase in SNR is merely in the single to double-digit range.

The enhanced and sustained plasma emission in the presence of microwaves can be attributed to several factors. The absorption of microwave energy by the plasma leads to sustainment and expansion of the plasma volume, which also enhances plasma emission owing to increased recombination and excitation processes. In addition, the continuous supply of energy provided by the microwaves may maintain the plasma in a non-equilibrium state for longer periods of time. The sustained plasma can provide more opportunities for atomic and molecular interactions, leading to enhanced emission intensities.

### Plasma temperature

The plasma temperature is commonly determined via spectrum analysis, with previous reports indicating that the vibration temperature of 12,000 K is reduced to approximately 2200 K using stoichiometric plasma spectrum measurement via a nitrogen second positive system (N2PS)^21, 30^. The vibration, rotation, and electron temperatures indicate physical characteristics of plasma that temporally change as the plasma shifts from equilibrium to nonequilibrium, making them challenging to evaluate. Microwaves have capturing atomic, ionic, and molecular emissions from sustained plasma easier, allowing for a successful evaluation of the electron temperature using the Saha–Boltzmann equation which is based on thermal equilibrium plasma. While the microwave-coupled plasma may not exist in a state of thermal equilibrium, the Saha–Boltzmann equation still offers a useful heuristic for estimating the electron temperature, as supported by multiple sources including Reference 38. Thus, we propose using the emissions from the air and ablation plasma interaction, which increase with microwaves, to easily and quickly approximate the plasma temperatures. This interaction leads to the emission of molecular N2PS, OH, and OI.

**Figure 5:**
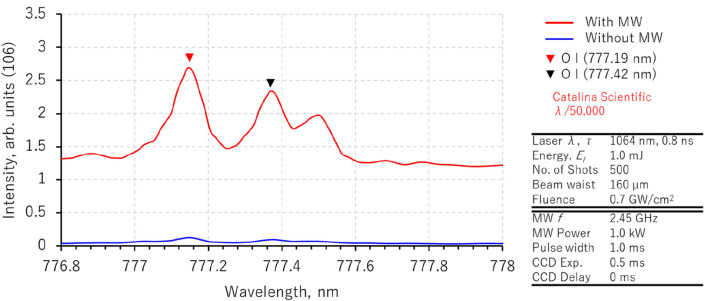
Comparison of OI triplet atomic emission in microwave-enhanced laser-induced breakdown spectroscopy (LIBS) with micro-laser.

In this study, we compared the plasma temperature characteristics using the electron temperature of the OI and discussed the temperature change of plasma expansion in space owing to microwave energy. We examined the microwave effect on electron temperature and its plasma sustainment in air. We performed high-resolution spectrometer measurements (λ/50,000) on two spectra of OI, 777.19 nm and 777.49 nm. Figure 5 illustrates the experimental results obtained via LIBS measurements using a microlaser with and without microwaves. These two OI spectra have different peak values depending on the presence or absence of microwaves; however, the microwaves also increase the continuum background light. We use spectrum comparison with the simulated spectra to calculate the electron temperature.

**Figure 6:**
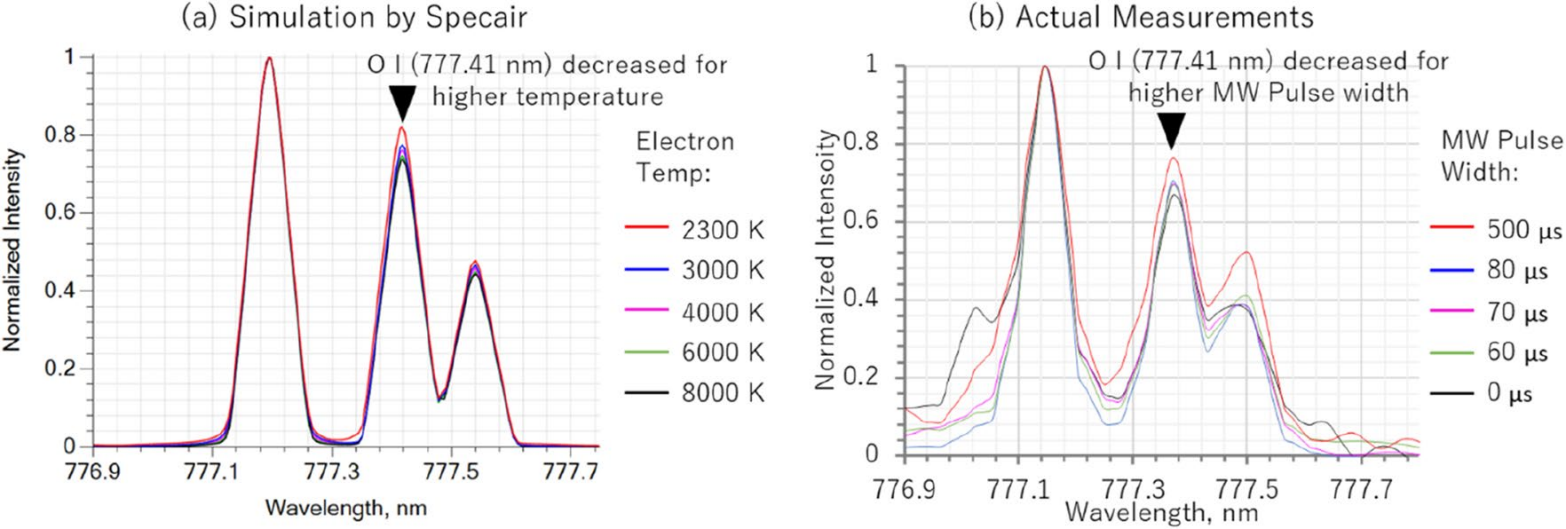
Temperature changes of OI spectrum and effect of microwaves pulse widths in microwave-enhanced laser-induced breakdown spectroscopy (LIBS) with micro-laser via (a) simulations in comparison to (b) actual measurements.

Figure 6a illustrate the simulated spectrum of the O band using synthetic spectra (SPECAIR 3.0, Spectral Fit, France)^18, 60^ as the electron temperature is increased from 2000 to 8000 K. SPECAIR calculates emission intensities based on energy level transitions, expressed by the Eq. 1:

1 $${\text{E}}_{{{\text{e}}^{\prime } {\text{v}}^{\prime } {\text{j}}^{\prime } }} = {\text{hc}}({\text{T}}_{{{\text{e}}^{\prime } }} + {\text{G}}_{{{\text{e}}^{\prime } }} \left( {{\text{v}}^{\prime } } \right) + {\text{F}}_{{{\text{e}}^{\prime } {\text{v}}^{\prime } }} \left( {{\text{J}}^{\prime } } \right) + {\text{st}}$$

In this equation, hc represents Planck's constant multiplied by the speed of light, while 'st' is a small term accounting for lambda doubling and spin splitting, as outlined in Reference 59. We observe a change in the peak value at 777.41 nm as the electron temperature increases, with the graph normalized to the 777.19 nm peak value. Figure 6b illustrates the measurement results of the OI spectrum under different microwave conditions. We also demonstrate the change in the 777.41 nm peak value, which is normalized to the 777.19 nm peak value, as the electron temperature increases. The microwave conditions are measured by changing the pulse from 0, 60, 70, 80, and 500 µs. Previous reports^21^ revealed that measurements of microwave absorbed power indicated exponential increase similar to the volume expansion of the ablation plasma within the first 100 µs. Beyond this period, both the absorbed power and volume expansion tend to stabilize or reach saturation levels; as such, our detailed measurements were conducted within the 100 µs timeframe. We note that the spectrum measured with a λ/50,000 spectroscope appears to be less smooth than the synthetic simulated spectrum. Regarding the normalized peak value at 777.41 nm, we’ve observed a trend of increasing values with increasing microwave input time. This trend aligns with the decreasing electron temperatures observed in the simulated spectra.

**Figure 7:**
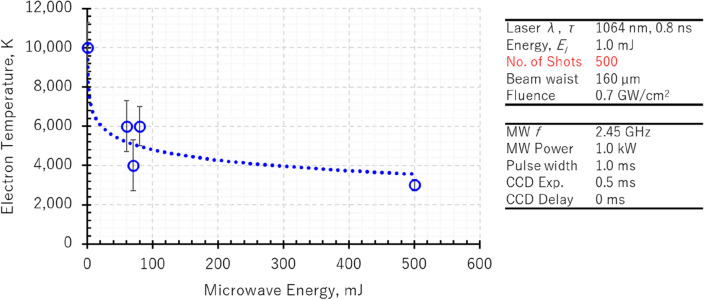
Comparison of electron temperature with microwave input period in microwave-enhanced laser-induced breakdown spectroscopy (LIBS) with micro-laser.

The effects of microwave pulse width on the electron temperature with 1.0 kW microwave are shown in Fig. 7. The electron temperature is approximately 10,000 K under the condition of no microwave input and drops to 6,000 K with microwave pulse width of 60 µs. From pulse width of 60–80 µs, the temperature fluctuates from 4000 to 6000 K. Subsequently, at microwave pulse width of 500 µs, the temperature slowly drops to 3000 K. The uncertainties of the measurements are around 1000 K, and all measurements were rounded to the nearest thousand.

We previously observed a striking similarity in the pattern of microwave power absorption to the pattern of temporal variations of electron temperature in air^24^. This observation suggests there is a correlation between the broadening of the microwave pulse and the time-variable alterations in electron temperature.

The increase in plasma volume due to microwave injection is 18 times larger which is accompanied by the electron temperature drops, after which the microwave injection sustained the non-equilibrium state of the plasma. Without microwaves, the plasma volume expansion and emission increase were driven by plasma relaxation and recombination effects, which result in a decrease in electron temperature and an increase in volume. This is distinctly different in the presence of microwaves, where the decrease in electron temperature does not result in a halt of plasma expansion. Instead, the microwaves cause a sustained level of electron excitation and activity, facilitating further plasma expansion.

**Figure 8:**
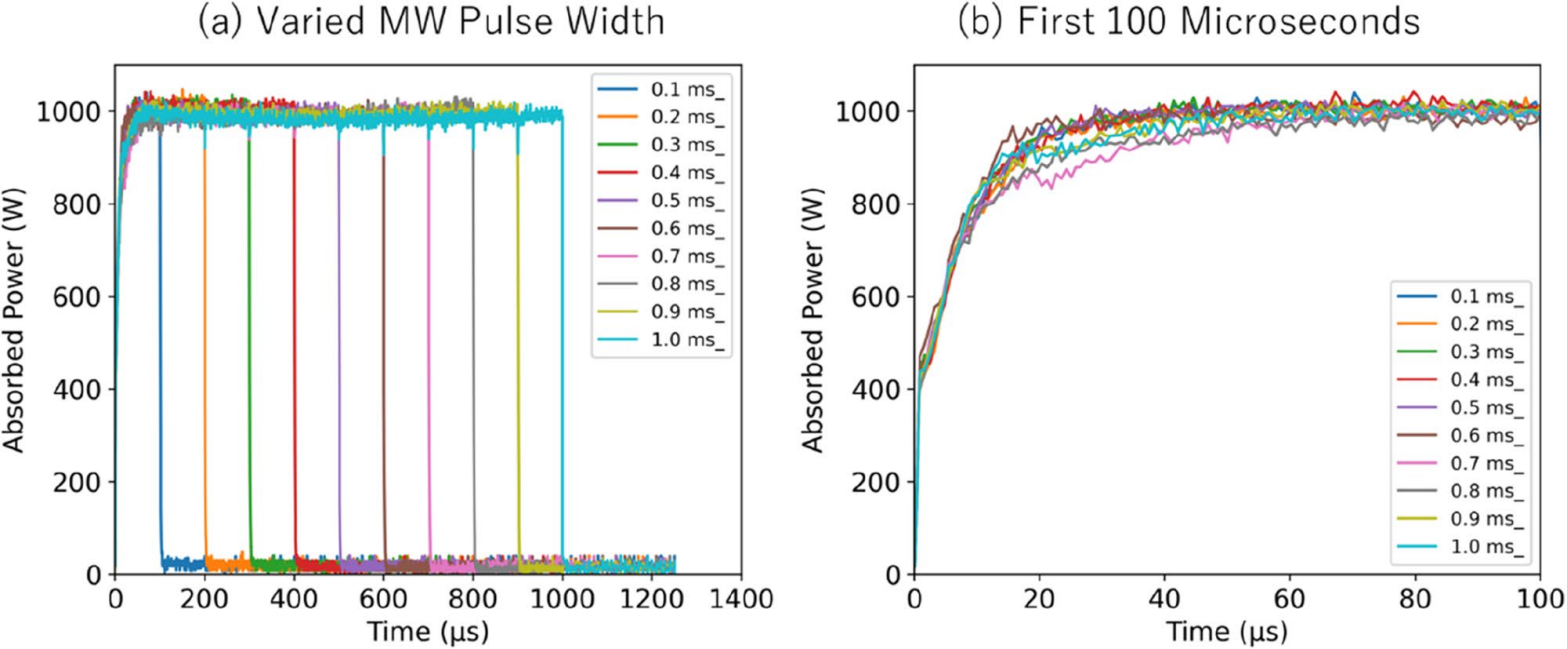
Measurements of temporal absorbed microwave power under two different conditions: (**a**) varying the microwave pulse width, and (**b**) within the first 80 μs.

Figure 8 illustrates the temporal absorbed microwave power for various microwave pulse widths ranging from 100 to 1000 μs. As shown in Fig. 8a, the microwave power absorbed by the laser ablation plasma consistently reached a maximum of over 98% for each pulse used. Figure 8b provides a more detailed view of the absorbed power during the initial 100 μs. Initially, the absorbed power is minimal due to the high density of the laser ablation plasma, which acts as a barrier to microwave propagation. However, this situation changes as time progresses; the absorbed power gradually increases, peaking at > 98% around the 60 μs mark. This trend—of minimal initial absorption followed by a gradual increase—is consistent across all varied microwave pulse widths. This consistency suggests a general relationship between microwave pulse width and temporal variations in microwave power absorption in laser ablation plasma.

## Discussions

Through this research, we have demonstrated the potential of utilizing microwaves to enhance the expansion of transient laser-induced ablation plasma generated by a Zr target. Our experimental design included a microwave helical antenna, serving as a near-field coupling system. The antenna delivered an approximate power density of 1.2 GW/m^2^ within a range of 0.1 mm from the target, leading to a remarkable enhancement of laser-induced ablation plasma. The power absorption initially remained minimal, peaking at 97.8% around 60 μs, corresponding to an absorbed power density of 7.9 GW/cm^3^. This power influx underlies the observed enhancement in plasma emission intensity and spatial volume by two to three orders of magnitude. The additional energy provided by the microwaves not only drives the excitation and ionization processes within the plasma, but also fuels its expansion.

Our experimental observations also revealed some challenges. We observed that the placement of the antenna in relation to the target has a bearing on the successful application of microwave-induced plasma expansion. Antenna-target distance and the enhancement factor did not seem to exhibit a direct correlation (refer to Supplemental Figs. 1 and 2), suggesting that other factors are be at play. However, adjusting the angle of the antenna relative to the laser propagation showed more consistent results. Smaller angular values resulted in higher emission enhancements. Hence, maintaining a consistent antenna-target distance of 0.1 mm and an antenna angle of 30 degrees from the laser propagation was key to achieving successful microwave-induced plasma expansion.

In terms of ablation formation on Zr oxide-based ceramic samples, we observed the formation of basin-like cavities. The application of microwaves led to a reduction in crater diameter and an increase in depth, indicating a potential influence of microwave energy on the volume of fumes produced during the ablation process. This observation marks a departure from previous studies that reported negligible effects of microwaves on ablation craters^21^. Both these observations have implications in fields such as toxic sample analysis and nuclear debris decommissioning.

Furthermore, we have investigated the temperature change of the plasma with and without the applied microwaves using the OI emissions. These emissions potentially arise from the ablation of the Zinc oxide layer or from interaction with the atmospheric air environment. Without microwave application, the electron temperature of the plasma was approximately 10,000 K. On the other hand, with microwave application, the electron temperature of the plasma initially dropped to 6000 K with microwave pulse width of 100 μs and then to 3000 K using higher microwave pulse width. This decrease in temperature is attributed to the expansion of the plasma, which subsequently increases interaction with atmospheric air environments. Expanding on this matter, a decrease in temperature should not be loosely associated with a reduction in volume as the electron temperature, electron density, and volume, are shaped by a variety of factors. This includes the influence of external forces, such as microwaves, on plasma. In our study, the microwaves not only sustained electron motion but also induced plasma expansion, leading to the observed temperature decrease. One likely reason for this decrease in temperature in an expanding plasma is the non-equilibrium nature of the plasma.

In previous study, we noted a decrease in the vibrational temperature in nitrogen molecules in air as the microwaves expanded the plasma, thereby increasing plasma-air interactions. We can thus confirm the formation of non-equilibrium plasma by the microwaves in both air plasmas and ablation plasmas. These findings demonstrate that the use of microwaves in laser-induced ablation has important implications for various applications, such as toxic sample analysis and nuclear debris decommissioning. The microwave-enhanced ablation plasma presented in this study could also be used for other applications such as material processing and micro/nano fabrication, where controlling plasma expansion and energy deposition is crucial. Overall, our study opens up new avenues for the development of more efficient and effective laser-based techniques for various applications.

## Conclusion

We developed a novel system that utilizes microwaves to enhance laser-induced ablation with microlaser. By superimposing microwaves on the laser ablation plasma, we induced breakdown by laser irradiation, and the ablated plasma was expanded and sustained in space by the microwaves. This expanded plasma was determined by the microwave energy and its injection time, independent of the initial laser conditions. Our results demonstrate that after breakdown, the plasma expands up to 18 times the initial size by absorbing microwaves.

We also investigated temperature changes in the plasma in the presence of microwaves. We wanted to emphasize that the electron temperature sustained in air differed significantly from the laser-induced ablation, as it was in a non-equilibrium state. In this state, the electron temperature was initially 10,000 K dropped to approximately 4000 K in approximately 100 µs. Subsequently, even with the application of microwaves, the electron temperature dropped to approximately 3000 K, and this state was maintained in space.

Our study provides new insights into the dynamics of plasma expansion and temperature changes in microwave-enhanced LIBS with a microlaser. The microwave can contribute to sustaining and expanding the plasma in air while decreasing the electron temperature. Our findings have important implications for various applications, including toxic sample analysis and nuclear debris decommissioning. The developed system may have significant potential for enhancing the sensitivity and selectivity of LIBS in various fields.

### Supplementary Information

Supplementary Figures.

## Data Availability

Data underlying the results presented in this paper are not publicly available at this time but may be obtained from the authors upon reasonable request by contacting Yuji Ikeda at yuji@i-lab.net.
