# Supplementary material for: Laser ablation plasma expansion using microwaves
Source: Sci Rep. 2023 Aug 25;13:13901. doi: 10.1038/s41598-023-41208-z (PMC10457374; doi:10.1038/s41598-023-41208-z)
Supplement: Supplementary file 1 — Supplementary Figures. [file 41598_2023_41208_MOESM1_ESM.docx]

Supplementary Figures


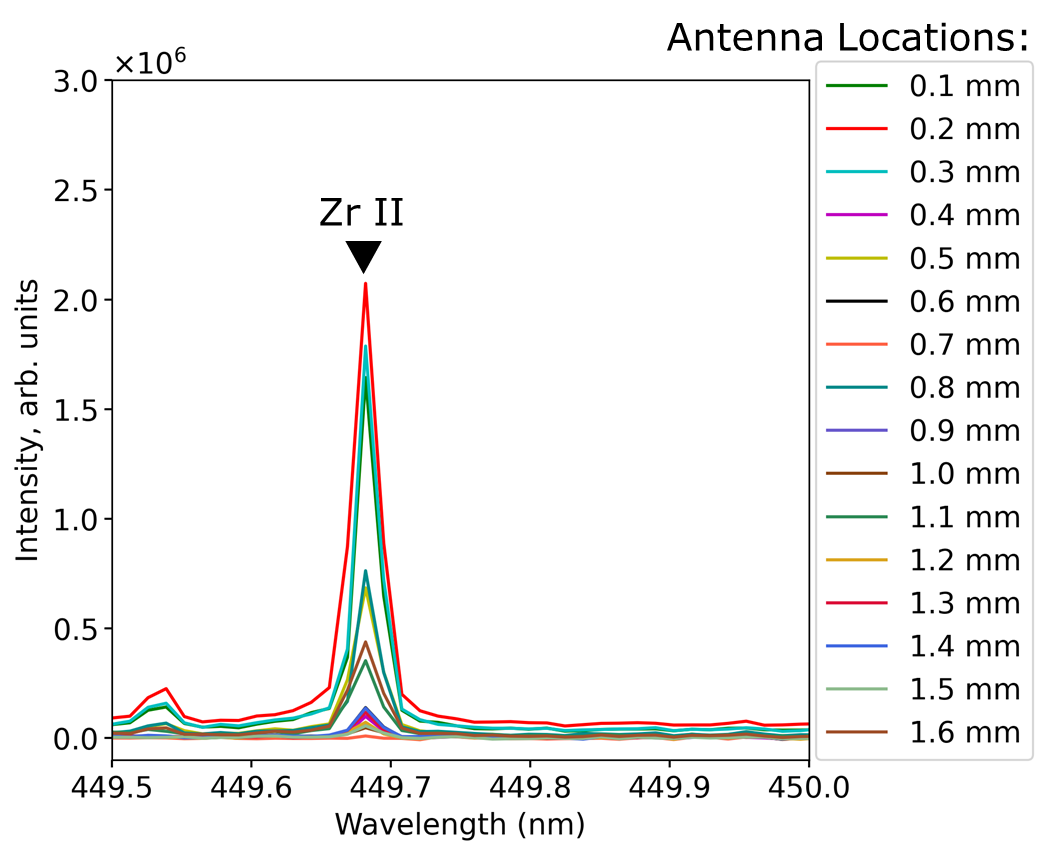


**Supplementary Figure 1**. Intensity emissions of Zr II at varied antenna locations from the target surface.


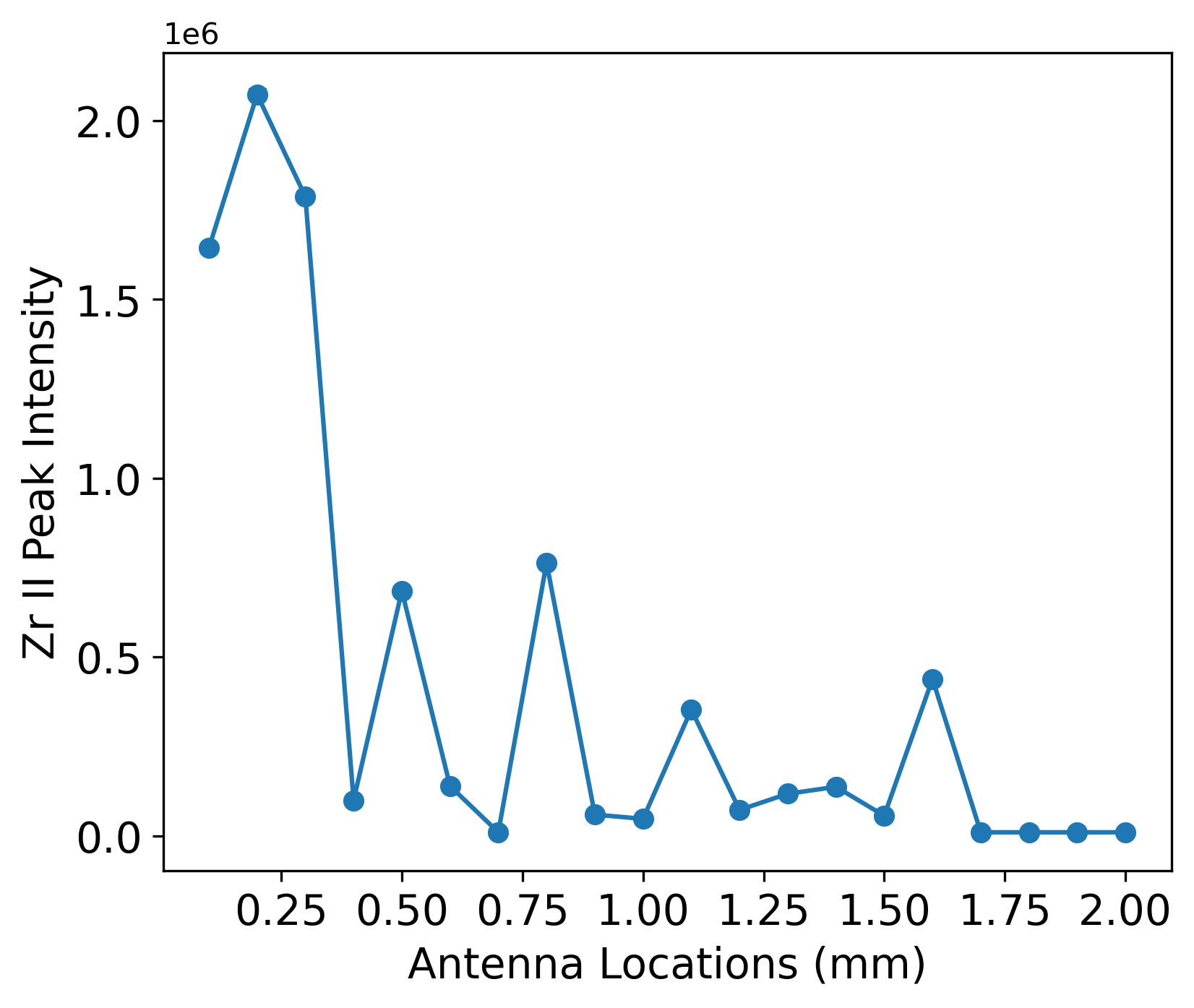


**Supplementary Figure 2**. Change in intensity emission peaks of Zr II at varied antenna locations from the target surface.
